# Unravelling the Phytotoxic Effects of Glyphosate on Sensitive and Resistant *Amaranthus palmeri* Populations by GC–MS and LC–MS Metabolic Profiling

**DOI:** 10.3390/plants12061345

**Published:** 2023-03-16

**Authors:** Ainhoa Zulet-Gonzalez, Karin Gorzolka, Stefanie Döll, Miriam Gil-Monreal, Mercedes Royuela, Ana Zabalza

**Affiliations:** 1 Institute for Multidisciplinary Research in Applied Biology (IMAB), Universidad Pública de Navarra, Campus Arrosadia s/n, 31006 Pamplona, Spain; 2 Leibniz Institute for Plant Biochemistry, Weinberg 3, 06120 Halle (Saale), Germany

**Keywords:** primary metabolism, phenylpropanoids, flavonoids, mode of action, EPSPS, non-targeted metabolomics, signalling molecules

## Abstract

Glyphosate, the most successful herbicide in history, specifically inhibits the activity of the enzyme 5-enolpyruvylshikimate-3-phosphate synthase (EPSPS; EC 2.5.1.19), one of the key enzymes in the shikimate pathway. *Amaranthus palmeri* is a driver weed in agriculture today that has evolved glyphosate-resistance through increased *EPSPS* gene copy number and other mechanisms. Non-targeted GC–MS and LC–MS metabolomic profiling was conducted to examine the innate physiology and the glyphosate-induced perturbations in one sensitive and one resistant (by *EPSPS* amplification) population of *A. palmeri*. In the absence of glyphosate treatment, the metabolic profile of both populations was very similar. The comparison between the effects of sublethal and lethal doses on sensitive and resistant populations suggests that lethality of the herbicide is associated with an amino acid pool imbalance and accumulation of the metabolites of the shikimate pathway upstream from EPSPS. Ferulic acid and its derivatives were accumulated in treated plants of both populations, while quercetin and its derivative contents were only lower in the resistant plants treated with glyphosate.

## 1. Introduction

Glyphosate is the most used herbicide worldwide, which specifically inhibits the 5-enolpyruvylshikimate-3-phosphate synthase (EPSPS) [1], a key enzyme from the shikimate pathway. Even though the glyphosate site of action is well established, it remains debatable how glyphosate kills plants. Currently, there are three hypotheses to explain plant death after herbicide application [2]. Firstly, insufficient production of aromatic amino acids (AAs), namely downstream metabolic pathway inhibition, as amino acids are vital for protein synthesis and plant growth. Secondly, toxicity can be related to substrate accumulation upstream from the inhibited enzyme. Finally, the possible involvement of side reactions induction has to be considered. Although physiological disturbances at the three points have been reported in glyphosate-treated plants, the specific importance of each physiological effect in the toxicity of the herbicide remains to be investigated.

The specific role of plant phenolics in the mode of action of the herbicide glyphosate has not been completely elucidated. Phenolic compounds are secondary metabolites, ubiquitous in plants with several important functions [3]. Among phenolic compounds, phenylpropanoids and flavonoids are synthesized from the aromatic AAs and explain the high metabolic flow through the shikimate pathway and its complexity in plants [4,5]. Although the inhibition of the synthesis of aromatic AAs by glyphosate has been extensively studied in plants, glyphosate effects beyond the shikimate pathway itself in plants are much less understood, with few studies focusing on phenolic metabolism.

The emergence of glyphosate-resistant (GR) weed populations due to the intensive and continuous use of the herbicide has become one of the most troublesome problems in weed management today [6]. As a global issue, herbicide resistance is a serious challenge for global food security [7]. One of the most damaging GR weed species is *Amaranthus palmeri* S. Wats, where the most common resistance mechanism is gene amplification of the target site *EPSPS* [8]. Although the exact resistance mechanism is in general well-established in GR populations, the exact resistant physiology of the populations and how it is affected by glyphosate remains to be elucidated.

Metabolomics is a multidisciplinary field combining analytical biochemistry with bioinformatics, providing knowledge to understand, monitor and identify metabolites whose levels are altered in response to abiotic or biotic stresses [9], such as herbicides [10,11,12]. Gas chromatography–mass spectrometry (GC–MS) and liquid chromatography–mass spectrometry (LC–MS) provide an ideal platform to screen many compounds simultaneously. With the help of bioinformatic tools such as the MeltDB 2.0-software [13], known and unknown compounds can be annotated, and relative abundances monitored. Metabolomics can be used as a functional tool to evaluate the effects of a specific herbicide, trying to obtain a metabolic overview of the treated plants.

Limited studies have employed metabolomics to evaluate the physiological effects of glyphosate in sensitive and resistant weeds [14,15,16]. To date, most of the metabolomics applications to GR plants have been dominated by the study of GR crops, especially transgenic maize [17,18]. The availability of a GR population with *EPSPS* gene amplification can be used to study additional effects of *EPSPS* overexpression on weed physiology. Moreover, the possible additional effects or different effects of glyphosate application on sensitive and resistant weeds in the metabolome can be detected by the use of GC–MS and LC–MS techniques.

The aim of this work was to characterize the innate differences (differences in the absence of glyphosate stress) and the glyphosate-induced physiological effects on two populations of *A. palmeri* (one glyphosate-sensitive (GS) and the other one glyphosate-resistant (GR)) using two types of metabolite profiling, as both methods work complementarily. First, non-targeted GC–MS profiling was performed to study smaller and polar compounds from primary metabolism. Second, LC–MS was performed to focus on the small- to medium-sized semipolar metabolites covering secondary metabolites.

## 2. Results and Discussion

In this study, non-targeted metabolic profiling was performed in two populations of *A. palmeri*, which were treated with two doses of glyphosate and compared to their respective nontreated checks. More than 80 metabolites were positively identified in the GC–MS analysis across all samples by their mass spectra fingerprints and retention index matches (Table S1). Around 8500 mass spectra features in positive mode and more than 4800 mass spectra features in negative mode were detected by LC–MS. Around 250 out of the 8500 features in the positive mode were annotated (Table S2).

### 2.1. Metabolic Comparison between Nontreated Plants of Sensitive and Resistant Populations

**Figure 1:**
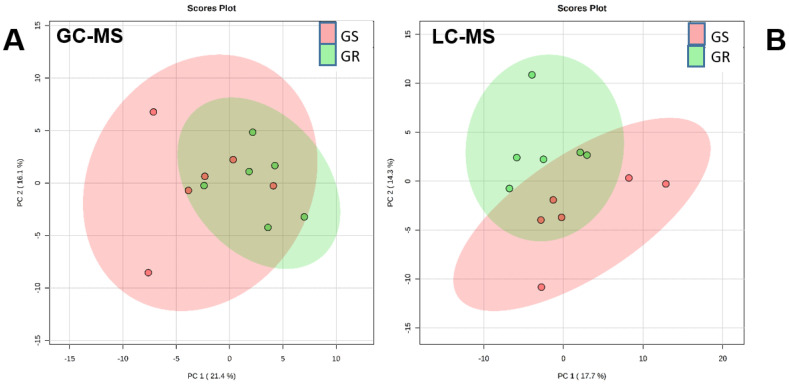
Principal component analysis (PCA) of metabolites detected by GC-MS (**A**) and LC-MS (**B**) in nontreated (control) glyphosate-sensitive (GS, in pink) and glyphosate-resistant (GR, in green) *Amaranthus palmeri* plants.

The availability of the biotype used in this study provides an opportunity to analyze the metabolome of a population with overexpression of *EPSPS* by comparison with a sensitive population. The metabolic profile between nontreated plants of both populations was compared by using GC–MS and LC–MS data. Principal component analysis (PCA) was performed with nontreated plants (control) with data from GC–MS (Figure 1A) and LC–MS (Figure 1B). The core plots of the first five principal components were visualized, and the two components that showed the greatest amount of differentiation between samples were chosen (PC1 and PC2).

PC1 and PC2 explained about 37.5% of the variance in GC–MS metabolites and about 32% in LC–MS metabolites. All points of both populations were located in the center in both GC–MS (Figure 1A) and LC–MS analysis, indicating that they were similar without glyphosate (Figure 1B). Moreover, no significant differences (*t*-Test, *p*-value ≤ 0.05) were detected for any of the metabolites detected by GC–MS or LC–MS. This lack of differences suggests that additional effects due to *EPSPS* overexpression at the metabolome level are not apparent. No other additional effects on the shikimate pathway beyond *EPSPS* overexpression was reported before for these two populations [19], but the results of the present study broaden the knowledge to metabolite profiles and demonstrate the lack of effects beyond the shikimate pathway. Other reports have shown after performing GC–MS analysis that nontreated glyphosate-sensitive and -resistant *A. palmeri* plants were very closely associated [15]. These results suggest that the metabolome is not affected by *EPSPS* overexpression in the GR population and that without glyphosate the increase in EPSPS protein would not change the abundance of metabolites in the shikimate pathway without showing any additional effect of the *EPSPS* gene amplification, as has been reported for other glyphosate-resistant *A. palmeri* populations [20,21].

### 2.2. Metabolic Profiling of Glyphosate-Treated Plants by GC–MS

**Figure 2:**
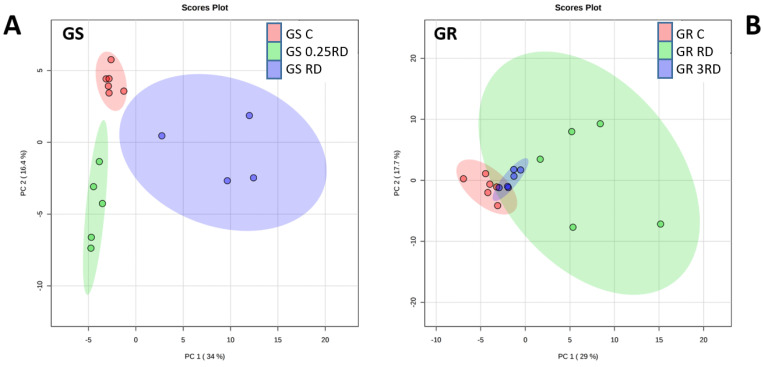
Principal component analysis (PCA) of metabolites detected by GC-MS in glyphosate-sensitive (GS; (**A**); **left**) and glyphosate-resistant (GR; (**B**); **right**) *Amaranthus palmeri* plants. Plants were not treated (control, C; in red) or treated with glyphosate at two doses: GS population with 0.25 RD (field recommended dose, in green) or RD (in purple); GR population with RD (in green) or 3 RD (in purple).

PCA was performed within each population (Figure 2) using the metabolites positively identified by GC–MS after treatment with two different doses of glyphosate. The doses applied to the GS population were the sublethal 0.25 times the recommended dose in the field (RD) and the lethal RD, while two sub-lethal doses were applied to GR population (RD and 3 RD).

In the GS population, different glyphosate doses showed a clear discrimination from one another when the plot of Component 1 was represented against Component 2 (Figure 2A). Component 1 seems to be related to the effect of the herbicide toxicity and clearly discriminates the highest and lethal glyphosate dose, RD, from the other non-lethal treatments. The presence or absence of glyphosate in each treatment can be discriminated by Component 2, separating control treatment (no herbicide) and the treatments of the two-glyphosate doses. In contrast, in the GR population, different treatments did not show a clear discrimination between them (Figure 2B). Control treatment and the lower glyphosate dose, RD, were very closed in the plot of Component 1 against Component 2, and the highest glyphosate dose was not clearly discriminated. Some of the top loadings of PCA analysis were similar for GS and for GR populations: in PC1 shikimate, S3P, dehydroshikimate, β-alanine and threonine and in PC2 aspartate, tyrosine, lysine and malate.

**Figure 3:**
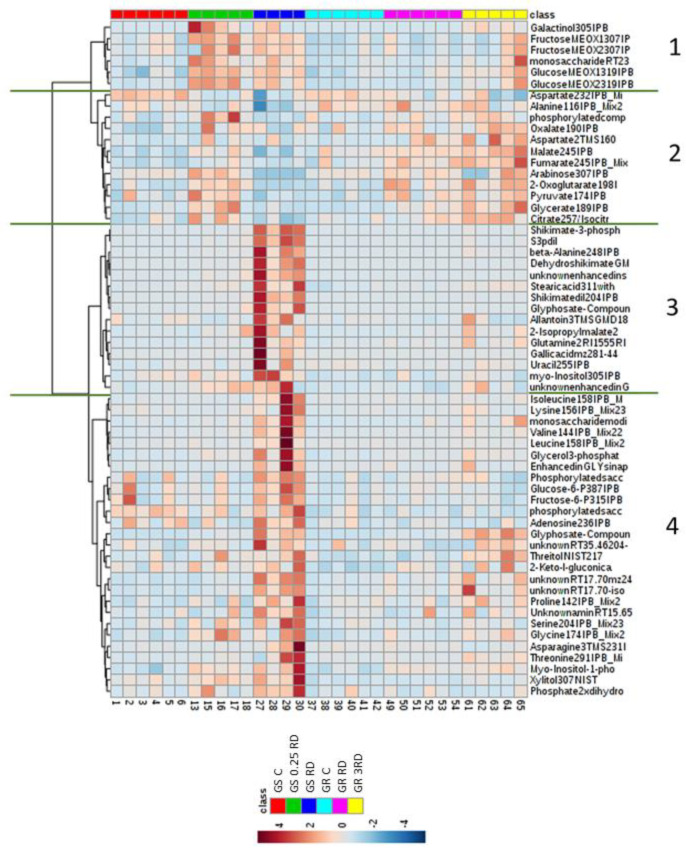
Heat map one-way hierarchical clustering (Clusters 1–4) of the ANOVA (Tukey) significantly different polar metabolites detected by GC–MS in glyphosate-sensitive (GS) and glyphosate-resistant (GR) *Amaranthus palmeri* plants. Plants were not treated (GS C and GR C) or treated with glyphosate at two doses: The GS population was treated with 0.25or 1 the field recommended dose (RD); the GR population was treated with 1 and 3 RD. The algorithm for heat map clustering was based on the Euclidean distance measure for similarity.

A heat map of metabolites that were significantly different in GS and GR after ANOVA (Tukey) was generated (Figure 3). The heat map showed differences in the metabolome profile between glyphosate doses but also differences between sensitive and resistant populations in their metabolic response to different doses. The 60 metabolites represented in the heat map were clustered by their abundance similarities and divided in 4 clusters after the treatments. Metabolites from Clusters 1 and 2 clearly separated from metabolites of Clusters 3 and 4.

After the application of glyphosate sublethal doses, metabolic changes were mainly detected in Clusters 1 and 2. There was an increase in the content of Cluster 1 metabolites, which included carbohydrates, such as glucose and fructose, after 0.25 DR in GS and 3 RD in GR populations. These two treatments also provoked an increase of Cluster 2 metabolite content, which was also slightly increased after treating the GR population with RD. This cluster was also formed by carbohydrates, organic acids and two amino acids (AAs), namely alanine and aspartate, suggesting that the primary metabolism would be altered when the toxicity is not very high.

However, after the lethal glyphosate dose (RD in the GS population), the metabolites that more intensely accumulated were clustered separately (Clusters 3 and 4). Although a slight effect on Cluster 1 was also detected, the most remarkable effects were observed in Clusters 3 and 4. Metabolites included in Cluster 3 were related to the shikimate pathway (S3P, shikimate, dehydroshikimate and gallic acid), and metabolites included in Cluster 4 were mainly AAs and carbohydrates.

### 2.3. Selected Primary Metabolites Detected by GC–MS

All metabolites identified by GC–MS were subjected to separate one-way ANOVA analysis within each population, in GS and in GR populations. A total of 17 metabolites out of 60 showed significant differences within the populations. They were primary metabolites belonging to different classes and were classified according to their relationship with the shikimate pathway and their characteristics: AAs, fatty acids, carbohydrates or Krebs cycle’s metabolites. Previous studies have also reported changes in almost all the metabolites determined by GC–MS [11,15,22,23]. Trying to decipher the physiological roadblock that results after the inhibition of EPSPS and eventually leads to plant death [24], we considered which possible cause (lack of end products of the shikimate pathway, substrate accumulation upstream from the EPSPS, or side effects of the inhibited pathway) was more related to glyphosate toxicity.

The lack of aromatic AAs as the cause of the death of the glyphosate-treated plants can be discarded. In fact, an increase in phenylalanine and tyrosine content was observed after glyphosate treatment in the GS population, even with the lethal dose (Figure S1) in concordance with other studies [25,26,27,28]. In addition to aromatic AAs, valine, leucine, alanine, asparagine, glutamine, threonine, lysine and serine content increased in the GS population after glyphosate treatment, and the effect was dose-dependent (Figure S1). A general increase in the free amino acid pool has also been widely described after glyphosate treatment [27,28,29], even with metabolomic techniques [22]. The observed accumulation of free amino acids in treated plants has been attributed to increased protein turnover [30], and indeed, a decrease in the content of total soluble protein was recently reported in glyphosate-treated *A. palmeri* populations [15]. In this way, the blockage of the shikimate pathway is immediately adjusted by the plant with a series of consequences (increase in the protein turnover) that lead to an increase in the free amino acid pool without any starvation of aromatic AAs. This imbalance was higher in the GS population after the lethal dose of herbicide, suggesting that this side effect would produce metabolic malfunctioning and important toxic consequences.

**Figure 4:**
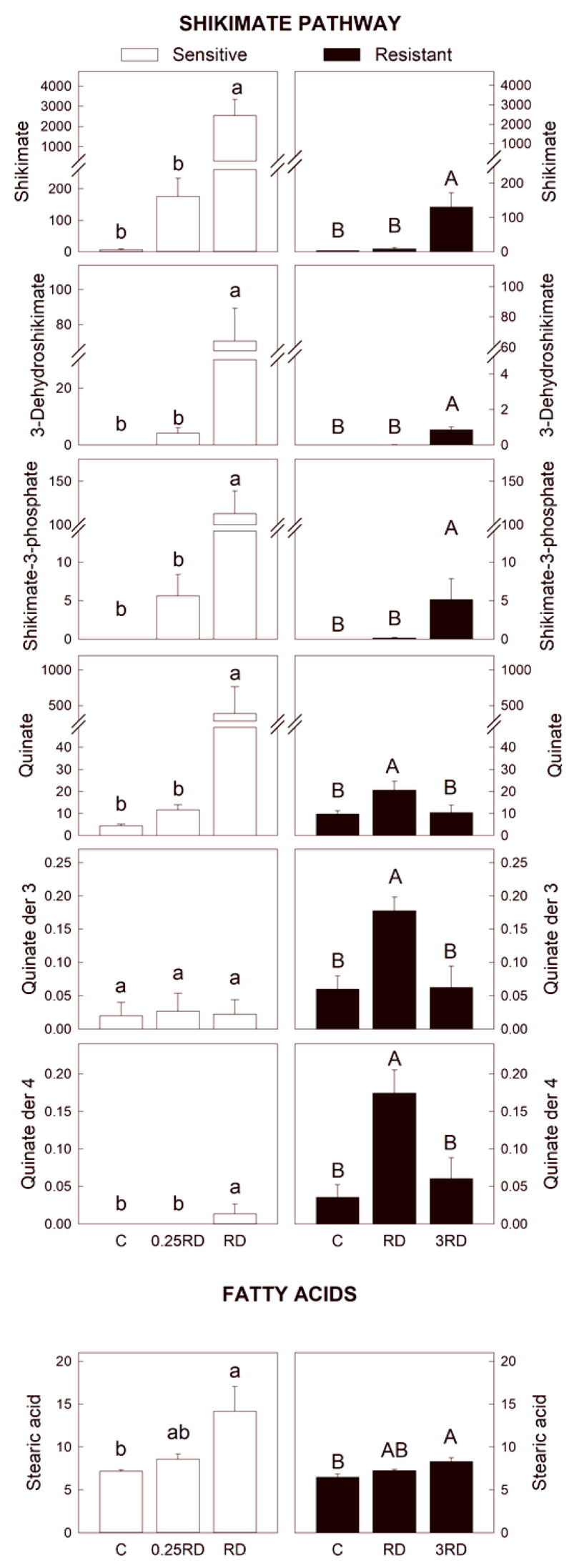
Selected primary metabolites detected by GC–MS in glyphosate-sensitive (GS) and resistant (GR) *Amaranthus palmeri* plants. Y-axis: normalized peak intensity: metabolites of the shikimate pathway and fatty acids. Plants were not treated (control, C) or treated with glyphosate at two doses. The GS population was treated with 0.25 or 1 the field recommended dose (RD); the GR population was treated with 1 and 3 RD; mean ± SE (n = 4–6). Different letters within each population indicate significant differences between treatments (*p*-value ≤ 0.05, Tukey).

The consideration of glyphosate toxicity by substrate accumulation revealed that the inhibition of the EPSPS by glyphosate provoked a striking accumulation of the metabolites upstream from the enzyme on the main branch of the shikimate pathway (shikimate, 3-dehydroshikimate and shikimate-3P) and of metabolites of lateral branches (quinate and quinate-derivative) (Figure 4). Quinic acid, gallic acid and protocatechuic acid, derivatives of the 3-dehydroshikimate, have been described to be accumulated in glyphosate-treated plants [28,31,32,33,34,35]. By far, the most highly accumulated metabolite after glyphosate is shikimate so that its content is commonly used as an indicator of glyphosate sensitivity [27,31,32,33]. Moreover, recently it has been demonstrated that several metabolic disturbances elicited after glyphosate treatments are mediated by shikimate accumulation, as glyphosate effects were mimicked in plants supplemented with exogenous shikimate [36].

In the GS population, the accumulation of metabolites upstream from EPSPS in the shikimate pathway showed a dose-dependent response (Figure 4). As a general pattern, the sub-lethal glyphosate dose did not provoke the accumulation of shikimate, 3-dehydroshikimate nor shikimate-3P. In GR plants, shikimate, 3-dehydroshikimate and shikimate-3P were accumulated only after 3 RD, and the accumulation detected was much lower than the accumulation detected in GS plants. Quinate and quinate derivatives were only induced in GR plants treated with the lowest dose, suggesting that their accumulation is not directly related to lethality. The EPSPS overexpression reduces the glyphosate effect in these GR plants, provoking a lower effect of the herbicide. The striking effect detected on GS plants after the lethal dose suggests substrate accumulation as one of the reasons for the lethality provoked by the herbicide.

Other adverse secondary effects on other pathways and processes are induced after the blockage of the shikimate pathway by the herbicide, so they can be considered as a possible cause of lethality in glyphosate-treated plants. The content of saturated stearic acid with 18 carbons (18:0) was increased in both populations (Figure 4), and the increase was dose-dependent. This effect of glyphosate on lipid composition can be considered as a physiological response to the stress induced by the herbicide. Indeed, the response of membrane lipids to different abiotic stresses can be considered as crucial in order to improve plant acclimatization ability to different environmental adversities [37].

**Figure 5:**
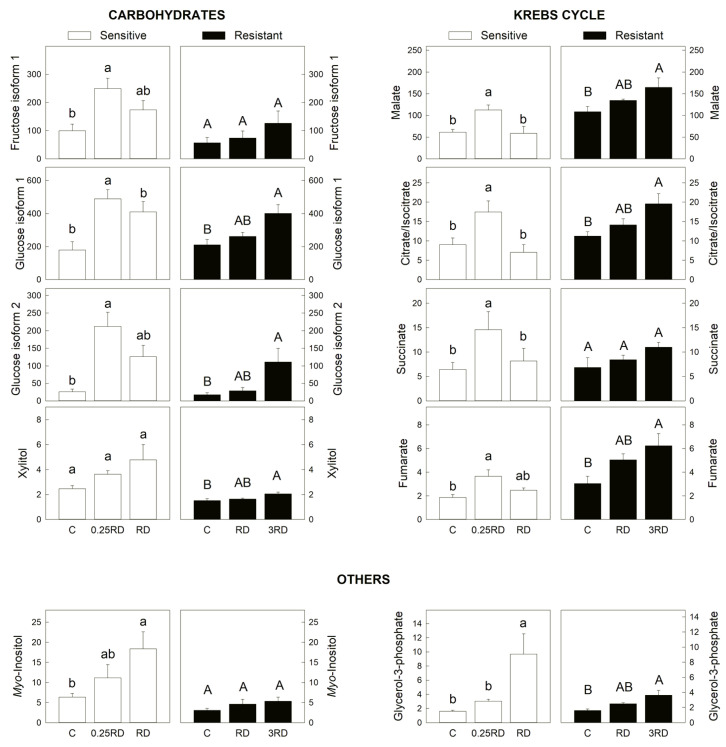
Selected primary metabolites detected by GC–MS in glyphosate-sensitive (GS) and resistant (GR) *Amaranthus palmeri* plants. Y-axis: normalized peak intensity: carbohydrates, Krebs cycle intermediates and others. Plants were not treated (control, C) or treated with glyphosate at two doses. The GS population was treated with 0.25× or 1× the field recommended dose (RD); the GR population was treated with 1× and 3× RD; mean ± SE (n = 4–6). Different letters within each population indicate significant differences between treatments (*p*-value ≤ 0.05, Tukey).

Glyphosate affected carbon metabolism by inducing changes in carbohydrate content and metabolites of the Krebs cycle. The glyphosate treatment on the GS population increased the relative content of fructose and glucose (carbohydrates) and malate, succinate, citrate/isocitrate and fumarate (organic acids) (Figure 5). Carbohydrate accumulation [26,27,38,39,40] and accumulation of the metabolites of the Krebs cycle [11,15] have been described before after glyphosate. It was proposed that carbohydrate accumulation in the leaves of the treated plants was due to a decrease in sink strength as the carbohydrates in the roots were not consumed [38] as growth was arrested. Indeed, the induction by glyphosate of the less energy-producing pathways of alternative oxidase and ethanol fermentation has been reported in the roots of several species [26,38,39] and in the roots of the populations used in this study [27].

The accumulation of carbohydrates and Krebs cycle metabolites confirmed that the primary metabolism is affected by the herbicide. Interestingly, the primary metabolism was more affected after the sub-lethal glyphosate dose and not after the lethal (RD) in the GS population. Similar changes at the level of many metabolites were detected in the GR population (Figure 5). Considering that the higher increases of metabolites were detected in both populations treated with sublethal treatments (0.25 RD in GS and 3 RD in GR), it can be proposed that the alteration of carbon metabolism is not directly related to the lethality provoked by the herbicide.

Myo-inositol (Figure 5) is a sugar-like carbohydrate produced by most plants that plays vital roles in plant biochemistry and physiology. Myo-inositol content was not affected in the GR population, while it was accumulated in the GS population treated with the lethal dose of glyphosate. Myo-inositol has been related to stress situations, for example by alleviating the salt-induced inhibition of physiological processes in *Malus hupehensis* [41].

### 2.4. Phenylpropanoids, Flavonoids and Signalling Molecules Detected by LC–MS

Among all metabolites annotated by LC–MS, metabolites related to phenylpropanoid and flavonoid pathways were used in this study in order to focus on the secondary metabolism after glyphosate treatment. In addition, two important signalling molecules (jasmonic acid and abscisic acid) were monitored.

**Figure 6:**
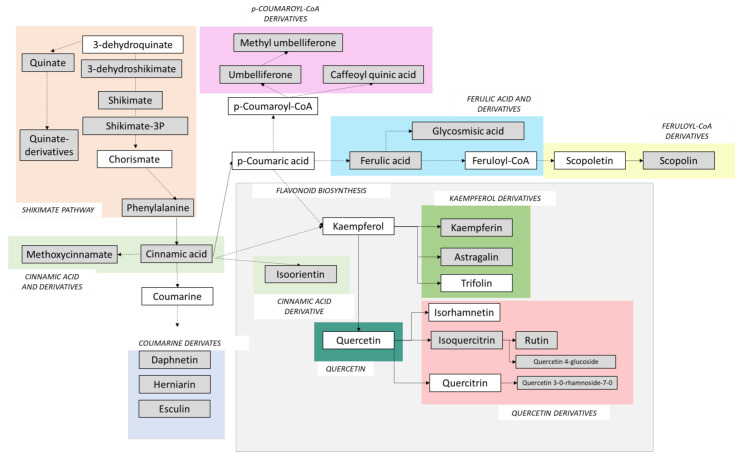
Simplification of the phenylpropanoid and flavonoid (squared in light grey) biosynthetic pathways and connection with the shikimate pathway. Metabolites in grey represent compounds found and annotated in this study. Metabolites in white were not detected or not positively annotated in this study. One-step reactions are represented by arrows with continuous lines. Reactions containing more than one step are represented by arrows with discontinuous lines. Different colors define chemical groups used for data plotting (Figure 7 and Figure 8).

**Figure 7:**
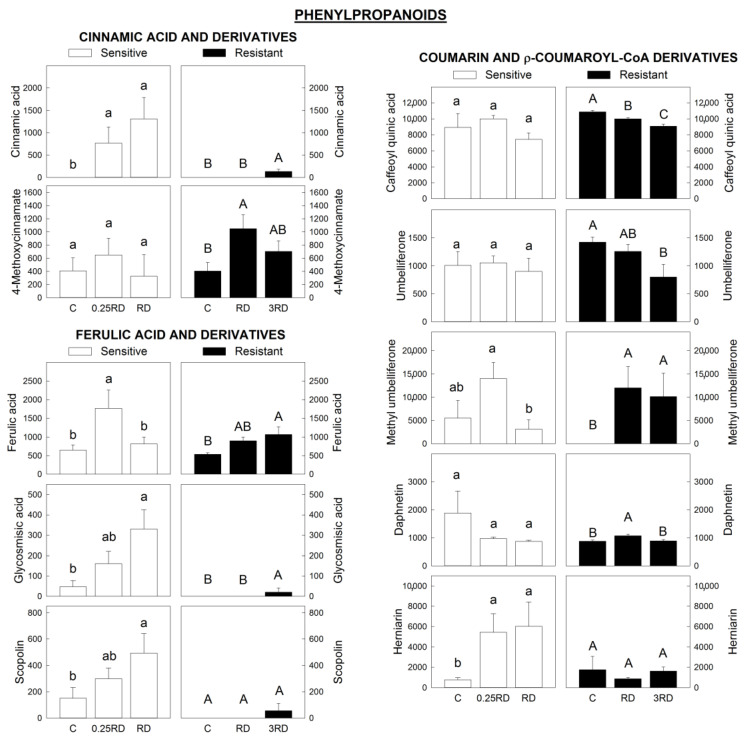
Some phenylpropanoids annotated by LC–MS in glyphosate-sensitive (GS) and-resistant (GR) *Amaranthus palmeri* plants. Y-axis: peak intensity: phenylpropanoids included are cinnamic acid and derivatives, ferulic acid and derivatives and derivative forms of coumarin and p-coumaroyl-CoA. Plants were not treated (control, C) or treated with glyphosate at two doses. The GS population was treated with 0.25 or 1 the field recommended dose (RD); the GR population was treated with 1 and 3 RD; mean ± SE (n = 6). Different letters within each population indicate significant differences between treatments (*p*-value ≤ 0.05, Tukey).

**Figure 8:**
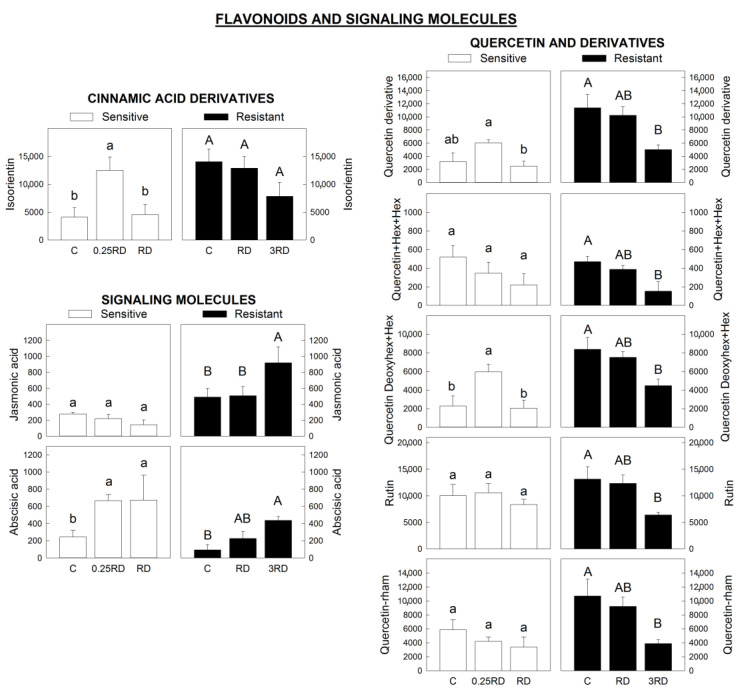
Flavonoids and signaling molecules annotated by LC–MS in glyphosate-sensitive (GS) and resistant (GR) *Amaranthus palmeri* plants expressed. Y-axis: peak intensity: flavonoids include cinnamic acid derivatives, quercetin and quercetin derivatives. Jasmonic acid and abscisic acid are the signaling molecules. Plants were not treated (control, C) or treated with glyphosate at two doses: The GS population was treated with 0.25 or 1 the field recommended dose (RD); the GR population was treated with 1 and 3 RD; mean ± SE (n = 6). Different letters within each population indicate significant differences between treatments (*p*-value ≤ 0.05, Tukey).

Most of the phenolic compounds originate from the phenylpropanoid and phenylpropanoid-acetate pathways. Lignins and coumarins, compounds with a C6-C3 core, are biosynthesized from the phenylpropanoid pathway. *P*-coumaric acid is a metabolite derived from the phenylpropanoid pathway that leads to the synthesis of C6-C3-C6 core molecules in the phenylpropanoid–acetate pathway, such as flavonoids and isoflavonoids. A schematic representation of the phenylpropanoid and the flavonoid biosynthetic pathways is included in Figure 6, where the metabolites detected in this study are shown in grey, and the shikimate pathway (Figure 4) is also included. Figure 7 and Figure 8 show the pattern of the annotated metabolites where significant differences within one population were detected.

Phenylpropanoids are involved in the protection machinery of plants due to their antioxidant ability [42,43]. Several studies confirm a general decrease in the production of phenylpropanoids (e.g., condensed lignins, anthocyanins, and flavonoids) following exposure to sublethal glyphosate doses in several plant species [44,45,46], but these metabolites have not been studied in depth. Figure 7 focuses on cinnamic acid, ferulic acid and their derivatives. While the contents of derivative forms of coumarin and p-coumaroyl-CoA did not show any homogeneous pattern after glyphosate, a dose-response effect of glyphosate was detected on cinnamic acid, ferulic acid and derivatives from ferulic acid in both populations. Phenylalanine ammonia lyase (PAL) enzyme catalyzes the deamination of phenylalanine to yield cinnamic acid. Glyphosate increases PAL activity in plants, as has been reported previously [28,45,47], which would explain the higher content of cinnamic acid (Figure 7). This suggests that the higher content of cinnamic acid would be diverted preferentially towards the ferulic acid branch. Ferulic acid accumulation has been proposed to be a reliable indicator of resistance to drought stress [48,49], indicating that sublethal doses of glyphosate elicit a general stress response in both GS and GR plants.

Interestingly, a different pattern between the two populations in some flavonoids and metabolites derived from them was detected (Figure 8). While in the GS population no significant changes were detected, in the GR population the content of quercetin and quercetin derivatives was diminished in the presence of glyphosate, showing a dose-response effect. Quercetins have been described to be very effective against reactive oxygen species [50], and the variations observed in quercetin derivatives after glyphosate in the GR population could be related in some way with the response to the herbicide-caused stress, although the reason for the lower content requires further studies. Indeed, the oxidant quenching efficiency could potentially complement the glyphosate resistance in another glyphosate-resistant *A. palmeri* population [15].

The content of two signalling molecules (jasmonic acid and abscisic acid (ABA)) was monitored. Jasmonic acid is responsible for inducing many defence responses in plants through the rapid production of effective compounds, such as alkaloids, terpenoids, antioxidants and phenylpropanoids [51]. However, it was only increased in the GR population treated with the highest dose of the herbicide, while no changes were detected in the GS population (Figure 8).

ABA is involved in mediating abiotic stress, dormancy and seed development. ABA content was increased after glyphosate treatment in both populations (Figure 8). In concordance with the results of this study, sublethal doses of glyphosate have been found to increase concentrations of ABA in soybean (*Glycine max*), suggesting that glyphosate, as other herbicides, is acting as an abiotic stressor [52]. The abscisic acid pathway may also be regulated by quercetin derivatives as suggested recently [50]. In this sense, it can be proposed that ABA content would be regulating the glyphosate effect on quercetin derivatives in both populations. To understand how glyphosate affects phytohormone biosynthesis and signalling, evidence from mechanistic studies that reveal clear causalities between glyphosate application, gene expression and phytohormone is urgently needed [53].

## 3. Materials and Methods

### 3.1. General Experiment Procedures

GC–MS analyses were performed on an Agilent instrument (GC: 6890 N, MS: 5975 MSD; Agilent Technologies; Santa Clara, CA, USA) with a ZB-5 Zebron Capillary column (30 m + 10 m Zebron, iD 0.25 mm, df 0.25 μm; Phenomenex, Torrance, CA, USA). LC–MS was performed by ultra-performance liquid chromatography (Waters Acquity UPLC equipped with a HSS T3 column (100 mm × 1.0 mm)) coupled to electrospray ionization quadrupole time-of-flight mass spectrometry (UPLC/ESI-QToF-MS) using a high-resolution MicrOTOF-QII hybrid quadrupole time-of-flight mass spectrometer (Bruker Daltonics, Bremen, Germany). All analytical products were LC–MS grade.

### 3.2. Plant Material and Glyphosate Treatments

*Amaranthus palmeri* seeds were originally collected from North Carolina (USA) [27,54]. The respective sensitivity and resistance of each population were tested by dose-response studies [27,54] and by in vivo shikimate assay [27]. Resistant (GR) individuals had a mean 47.5-fold increase in the number of copies of the *EPSPS* gene in comparison to sensitive individuals (GS) [27]. Seeds were surface-sterilized, germinated and then transferred to 2.7-L tanks in a phytotron where seedlings were grown in aerated hydroponic culture, as described previously [27]. Glyphosate treatments were applied after three weeks of hydroponic culture. Each population was treated with a low and a high glyphosate dose [55] relative to the recommended field dose (RD) of 0.84 kg ae ha^−1^ [56]. GS plants were treated with 0.25 times RD (0.25 RD) and RD, while GR plants were treated with RD and 3 times RD (3 RD). The commercial glyphosate Fortin Green^®^ (Key, Tárrega, CAT, Spain) was used; 2.52 mL of the herbicide solution per tank (4.59 dm^2^) was sprayed using an aerograph (model Definik, Sagola, Vitoria-Gasteiz, EUS, Spain) connected to a compressor (model Werther, Breverrato, Italy; 60 W, 10 L m^−1^, 2.5 bar) inside a flow cabinet (Figure S2). Nontreated plants were sprayed with tap water using the same aerograph and compressor. The experiment was performed twice. Although results were corroborated, only results from the first experiment are shown as they were similar.

Three days after herbicide treatment, leaves were sampled, frozen in liquid N_2_ and stored at −80 °C. Leaves of the same plant were collected together in an individual vial, and each vial behaved as a replicate. Different plants behaved as biological replicates. Samples were ground into powder using a Retsch mixer mill (MM200, Retsch, Haan, Germany), and the amount of tissue needed for each analytical determination was separated.

### 3.3. Metabolite Extraction for GS–MS and LC–MS

Each sample (0.1 g of leaf sample) was extracted with 500 µL of extraction solution (80% methanol with 100 µM ribitol, 5 µM kinetin, 5 µM biochanin A and 5 µM IAA-Valine). All reagents were LC–MS grade. Griding beads (Zirkonia/Glass beads, 0.5 mm, Biospec Products) were added to each sample in Eppendorf tube 2 mL screw-top vials (Sarstedt); extraction solution was added afterwards, and samples were homogenized with a tissue homogenizer (Precellys 24, lysis and homogenization, Bertin Instruments, Montigny-le-Bretonneux France) for 2 × 45 s at 6500 rpm. Samples were placed on a shaker (Ika^®^ Vibrax VXR basic, IKA^®^-Werke GmbH & Co. KG, Staufen, Germany) for 10 min at 1000 rpm in an ultrasonic bath (Sonorexdigitec, Bandelin, Germany) in floaters for 10 min and then replaced in the shaker for 10 min at 1800 rpm. Samples were centrifuged at max. speed in a table-top centrifuge for 15 min (RT)(Centrifuge 5425 R, Eppendorf AG). Pellets were reextracted again as before, and the supernatants of the first and second extraction were placed in a 1.5 mL reaction tube (Eppendorf) and vortexed.

### 3.4. Non-Targeted Metabolic Profiling by GC–MS

For GC–MS, a 50 µL-aliquot of the extract was transferred to a new Eppendorf tube and evaporated at 37 °C (SpeedVac). Methoxyamine hydrochloride was dissolved in 1 mL pyridin by shaking for 30 min (40 mg). An aliquot (25 µL) of this solution was added to each sample and incubated 1.5 h at 40 °C under constant shaking. BSTFA (1 mL) was mixed with 100 µL of RI-Mix (alkane standard, prepared as in Gorzolka et al. [57]), and 25 µL of that mixture was added to the sample. The samples were then incubated for another 30 min at 40 °C in the shaker and transferred to a GC–MS vial.

Test derivatization and analyses showed that some samples demonstrated several very high peaks. For proper metabolite profiling and with the aim of identifying and quantifying as many compounds as possible in the linear dynamic response range, all samples were analyzed in two dilutions according to Gorzolka et al. (2012) [57].

The raw data were converted to cdf-files by the MassHunter Qualitative Analysis software B.07.00 (Agilent Technologies) and uploaded to the MeltDB 2.0 software. In MeltDB, metabolite profiling was performed with peak picking thresholds of SN = 5 and FWHM = 5 using the warped algorithm. Retention indices were annotated manually. Identification of metabolites by mass spectra similarity was performed in MeltDB based using in-house reference libraries from analytical reference standards. Metabolite annotations are based on NIST11 and GMD database MS similarity search as well as on observations on typical MS fragmentation patterns. Each compound was reviewed for proper annotation and alignment. Metabolites that were not detected properly were quantified manually in DataAnalysisQedit (ChemStation D0.2, Agilent Technologies).

Peaks were quantified on the peak area of characteristic ion traces and normalized on ribitol as internal standard to compensate for device performance variation. Data were exported to Excel for further statistics as well for import in MetaboAnalyst “https://www.metaboanalyst.ca/ (accessed on 15 February 2020)”.

### 3.5. LC–MS

Extracts were mixed 80% with 20% water with 0.1% formic acid (Solvent A). Samples were stored overnight at −20 °C and centrifuged for 15 min before pipetting the supernatant into the LC-Vials. Ultra-performance liquid chromatography (Waters Acquity UPLC equipped with a HSS T3 column (100 mm × 1.0 mm)) coupled to electrospray ionization quadrupole time-of-flight mass spectrometry (UPLC/ESI-QToF-MS) was performed using a high-resolution MicrOTOF-QII hybrid quadrupole time-of-flight mass spectrometer (Bruker Daltonics). Data were acquired in centroid mode with the following MS instrument settings for positive mode: nebulizer gas: nitrogen, 1.6 bar; dry gas: nitrogen, 6 L/min, 190 °C; capillary, 4000 V; end plate offset: 500 V; funnel 1 radio frequency (RF): 200 Volts peak-to-peak (Vpp); funnel 2 RF: 300 Vpp; in-source collision-induced dissociation (CID) energy: 0 eV; hexapole RF: 100 Vpp; quadrupole ion energy: 3 eV; collision gas: argon; collision energy: 5 eV; collision cell RF: 300 Vpp; transfer time: 70 μs; prepulse storage: 5 μs; pulser frequency: 10 kHz; and spectra rate: 3 Hz. Data were processed by MetaboScape 4.0 software (Bruker, Germany). Metabolites were identified by their retention time and accurate mass compared to analytical standards.

### 3.6. Statistical Analysis

Analyses were performed using 4–6 biological replicates using samples from different individual plants.

The normalized intensities of metabolites identified by GC–MS were analyzed by multivariate statistical analysis using MetaboAnalyst 4.0 software “https://www.metaboanalyst.ca (accessed on 15 February 2020)”. The metabolomics data were auto-scaled before multivariate analysis, and only metabolites identified were used.

First, differences between GC–MS values and metabolites identified in the positive mode of LC–MS of nontreated plants of both populations were evaluated using PCA and Student’s *t*-test (*p* ≤ 0.05).

Second, PCA was conducted on each population to study the effect of the herbicide on GC–MS values. The top loadings of principal Components 1 and 2 were selected, and for these metabolites, differences between treatments within each population were evaluated by performing one-way ANOVA with a multiple-comparison adjustment (Tukey) at *p* ≤ 0.05 with IBM SPSS Statistics. After that, hierarchical cluster analysis of all the significantly different metabolites (ANOVA) was performed and visualized using heat maps.

Third, for LC–MS identified metabolites, the effect of glyphosate on phenylpropanoid and flavonoid biosynthetic pathways between treatments within each population was evaluated using one-way ANOVA with a multiple-comparison adjustment (Tukey) at *p* ≤ 0.05. 

## 4. Conclusions

Overall, the innate cellular physiology detected by GC–MS and LC–MS was not different between resistant and susceptible plants, which implies that additional effects due to shikimate pathway perturbation are not apparent.

GC–MS profiling revealed that glyphosate-induced plant death was not due to the lack of aromatic AAs, as all AAs were accumulated in the treated plants, including aromatic AAs. So, lack of end products of the pathway can be discarded as the main reason of the lethality of the herbicide.

Lethal doses of glyphosate elicited accumulation of the metabolites of the shikimate pathway upstream from EPSPS (shikimate, 3-dehydroshikimate and shikimate-3P) and resulted in perturbations in carbon metabolism (accumulation of carbohydrates and metabolites of the Krebs cycle) in the sensitive plants. These results suggest that substrate accumulation and additional effects are related to plant death after herbicide application.

While primary metabolites were almost not affected in treated-GR plants, phenylpropanoids and flavonoids were more affected in both populations. Ferulic acid and its derivatives were accumulated in treated plants of both populations, while quercetin and its derivative contents were lower only in the resistant plants. The increase of the plant hormone ABA was detected in both populations, while jasmonic acid only increased in the GR population.

## Data Availability

All relevant data are within the manuscript.

## Supplementary Materials

The following supporting information can be downloaded at: https://www.mdpi.com/article/10.3390/plants12061345/s1, Table S1. Metabolites identified and fragment ions (*m*/*z*) in *Amaranthus palmeri* GS and GR plants by GC–MS. Table S2. Metabolites identified in *Amaranthus palmeri* GS and GR plants by LC–MS. Figure S1. Amino acids detected by GC–MS. Figure S2. Treatment application using an aerograph connected to a compressor and plant status. Reference [58] is cited in the supplementary materials.
