# Supplementary material for: Unravelling the Phytotoxic Effects of Glyphosate on Sensitive and Resistant Amaranthus palmeri Populations by GC–MS and LC–MS Metabolic Profiling
Source: Plants (Basel). 2023 Mar 16;12(6):1345. doi: 10.3390/plants12061345 (PMC10058430; doi:10.3390/plants12061345)

Table S1. Metabolites identified and fragment ions (m/z) in *Amaranthus palmeri* GS and GR plants by GC-MS. ID level: Compound was Identified by Authentic Reference standard (1), MS data base search (2) or database-search with striking hits; thus interpretation of MS peaks was done (3).

Metabolites	Abbreviation in Heatmap	ID level	Fragment Ions (m/z)
2-Aminoadipate	2-Aminoadipate	1	260
2-Isopropylmalate	2-Isopropylmalate	1	275
2-Keto-l-gluconic acid	2-Keto-l-gluconic acid	1	292
2-Oxoglutarate	2-Oxoglutarate	1	198
Adenine	Adenine	1	264
Adenosine	Adenosine	1	236
Adenosine-5-monophosphate	Adenosine-5-MP	2	169
Alanine	Alanine	1	116
Allantoin	Allantoin	2	189, 259, 359
Arabinose	Arabinose	1	307
Arginine	Arginine	1	157
Asparagine	Asparagine	1	231
Aspartate 1	Aspartate 1	1	160
Aspartate 2	Aspartate 2	1	232
Benzoate	Benzoate	1	179
Benzoate derivate	Benzoate der.	3	179, 245
beta-Alanine	beta-Alanine	1	248
Caffeate 1	Caffeate 1	1	219
Caffeate 2	Caffeate 2	1	396
cis-Aconitate	cis-Aconitate	1	229
Citrate / Isocitrate	Citrate	1	257
Dehydroshikimate	Dehydroshikimate	2	296, 402, 417 (386)
Ethanolamine	Ethanolamine	1	174
Ferulic acid / Isoferulic acid	(Iso-)Ferulate	1	338
Fructose 1	Fructose 1	1	307
Fructose 2	Fructose 2	1	307
Fructose-6-Phosphate	Fructose-6-P	1	315
Fumarate	Fumarate	1	245
Galactinol	Galactinol	1	305
Gallic acid	Gallic acid	2	281, 443 (458)
Glucose 1	Glucose 1	1	319
Glucose 2	Glucose 2	1	319
Glucose-6-phosphate	Glucose-6-P	1	387
Glutamate	Glutamate	1	246
Glutamine 1	Glutamine 1	1	RI 1488
Glutamine 2	Glutamine 2	1	RI 1555
Glutaric acid	Glutaric acid	1	261
Glycerate	Glycerate	1	189

Glycerol 3-phosphate	Glycerol 3-P	2	357
Glycine	Glycine	1	174
Glyphosate-Compound RT 19.00	Gly-Cmp 1	3	282
Glyphosate-Compound RT 22.40	Gly-Cmp 2	3	212
Glyphosate-Compound RT 23.00	Gly-Cmp 3	3	155
Hexadecanoic acid	Hexadecanoate	2	313
Isocitric lactone	Isocitric lactone	2	303
Isoleucine	Isoleucine	1	158
Leucine	Leucine	1	158
Lysine	Lysine	1	156
Malate	Malate	1	245
Maleic acid	Maleate	2	245
Methionine	Methionine	1	176
monosaccharide RT 23.20	Monosaccharide 1	3	204
Monosaccharide with modification	Monosaccharide 2	3	319
myo-Inositol	*myo*-Inositol	1	305
myo-Inositol-1-phosphate	*myo*-Inositol-1-phosphate	1	318
Octadecadienoic acid	Octadecadienoic acid	2	337
Octadecanoic acid	Octadecanoic acid	2	341
Octadecenoic acid	Octadecenoic acid	2	339
organic acid RT 23.07 galactaric acid	Organic acid 1	3	333
organic acid RT 23.45 Glucaric acid	Organic acid 2	3	333
Oxalate	Oxalate	1	190
Phenylalanine	Phenylalanine	1	192
Phosphate + 2x dihydroxypropyl group	*Ukn P+dihydroxypropyl*	3	357-503-445-299
Phosphorylated saccharide RT 27.25	Saccharide-P 1	3	387
phosphorylated saccharide RT 29.75	Saccharide-P 2	3	387
Proline	Proline	1	142
Pyrophosphate 4TMS	Pyrophosphate 4TMS	2	451
Pyruvate	Pyruvate	2	174
Quinate	Quinate	1	345
Quinic acid derivative (glycosylated?) RT 31.40 mz	Quinate derivate 1	3	255-204-361 (345)
Quinic acid derivative (glycosylated?) RT 31.80 mz	Quinate derivate 2	3	255-204-361 (345)
Quinic acid derivative (glycosylated?) RT 32.10 mz	Quinate derivate 3	3	255-204-361 (255)
Quinic acid derivative (glycosylated?) RT 32.40 mz	Quinate derivate 4	3	255-204-361 (345)
Ribitol	Ribitol	1	217
Serine	Serine	1	204

Shikimate	Shikimate	1	204
Shikimate-3-phosphate	Shikimate-3-phosphate	2	483
Sinapic acid derivate	Sinapic acid derivate	3	227, 301, 329 (344)
Stearic acid	Stearic acid	2	311, 453
Succinate	Succinate	1	247
Sucrose	Sucrose	1	361
Threitol	Threitol	2	217
Threonic acid	Threonic acid	2	292
Threonine	Threonine	1	291
Tyramine	Tyramine	1	174
Tyrosine	Tyrosine	1	280
Uracil	Uracil	2	255
Valine	Valine	1	144
Xylitol	Xylitol	1	307
Xylitol or similar monosaccharide / alcohol	Xylitol-like	3	307
Unknown 1	*Unknown 1*		*245, 308, 410, 424*
Unknown 2	*Unknown 2*		*224, 296, 386*
Unknown amin RT 15.65	*Ukn amin 1*		*174, 156*
unknown amin RT 17.30	*Ukn amin 2*		*174*
unknown phosphorylated compound RT 32.90	*Ukn-P*	*3*	*169, 450, 384*
unknown RT 17.70	*Ukn RT 17.70 1*		*275*
unknown RT 17.70	*Ukn RT 17.70 2*		*244, 275*
unknown RT 18.60	*Ukn RT 18.60*		*230, 257, 359, 374*
unknown RT 22.40	*Ukn RT 22.40*		*424*
unknown RT 22.70	*Ukn RT 22.70*		*155, 271, 299, 447*
unknown RT 25.30	*Ukn RT 25.30 1*		*204, 210, 235*
Unknown RT 25.30	*Ukn RT 25.30 2*		*204, 217, 235*
unknown RT 33.60	*Ukn RT 33.60*		*307, 375, 219, 714*
unknown RT 34.90	*Ukn RT 34.90*		*307, 375, 219, 714*
unknown RT 35.12	*Ukn RT 35.12*		*307, 375, 219, 714*
unknown RT 35.46	*Ukn RT 35.46*		*204, 217, 361, 597*
unknown steroid-like compound isomer 1 RT 28.40	*Steroid-like 1*	*3*	*245, 360, 430, 520, 592*
unknown steroid-like compound isomer 2 RT 28.55	*Steroid-like 2*	*3*	*245, 360, 430, 520, 592*

Table S2. Metabolites identified in *Amaranthus palmeri* GS and GR plants by LC-MS. Confidence level of annotation (According to Matern et al., 2019) is as follows: 1, verified with mass, retention time, and CID spectrum of a commercial standard or a synthesized standard; 2, putatively annotated compounds based on CID spectrum interpretation, data base hits, and literature; 3, possible structure or metabolite family based on hits in data bases; 4, unknown.

			Quantifier ion		
**Annotation**	**Elemental composition**	**RT [min]**	**Type**	**m/z meas.**	**Ann. level**
Pipecolinic acid	$C_6H_{11}NO_2$	0.37	[M+H]+	130.09266	2
L-Histidine	$C_6H_9N_3O_2$	0.37	[M+H]+	156.0744	1
L-Proline	$C_5H_9NO_2$	0.4	[M+H]+	116.07113	1
Ribulose 1,5-bisphosphate	$C_4H_4O_7P_2$	0.42	[M+H]+	226.95123	1
Cytosine	$C_4H_5N_3O$	0.43	[M+H]+	112.05308	1
D-Ribose 5-phosphate	$C_5H_{11}O_8P$	0.43	[M+K]+	268.9814	1
L-Valine	$C_5H_{11}NO_2$	0.43	[2M+Na]+	257.14615	1
unknown ubiquitous metabolite	$C_4HO_2PS_4$	0.44	[M+H]+	240.86606	4
D-Xylitol	$C_5H_{11}NaO_5$	0.45	[M+Na]+	175.05737	1
Betaine	$C_5H_{11}NO_2$	0.47	[2M+H]+	235.16485	1
Trigonelline	C_7H_7NO2	0.47	[M+H]+	138.05513	1
Nicotinic acid	$C_6H_5NO_2$	0.48	[2M+H]+	247.07745	1
D-Mannitol	$C_6H_{14}O_6$	0.48	[M+CO_2H_2+H]+	229.09487	1
Betaine	$C_5H_{11}NO_2$	0.48	[M+H]+	118.08674	1
D-Sucrose	$C_{12}H_{22}O_{11}$	0.5	[M+Na]+	365.10311	1
Fructose/Glucose	$C_6H_{12}O_6$	0.5	[M+Na]+	203.0513	3
L-Asparagine	$C_4H_8N_2$O3	0.5	[2M+Na]+	287.10002	1
Adenine	$C_5H_5N_5$	0.5	[M+H]+	136.06184	1
Quinate	$C_7H_{12}O_6$	0.51	[M+Na]+	215.05107	1
L-Arginine	$C_6H_{14}N_4O_2$	0.51	[M+CH_2O_2Na]+	243.11056	1

L-Glutamine	$C_5H_{10}N_2O_3$	0.54	[M+H]+	147.07496	1
Glutamic acid	$C_5H_9NO_4$	0.55	[M+H]+	148.06083	1
Inosine 5'-monophosphate	$C_{10}H_{13}N_4O_8P$	0.57	[M+H]+	349.06017	1
L-Ureidosuccinic acid	$C_4H_7NO_4$	0.62	[M-CHNO+H]+	134.04472	1
Glutathione	$C_{10}H_{17}N_3O_6S$	0.64	[M]+	307.08285	1
Citric acid	$C_6H_8O_7$	0.64	[M+K]+	230.99227	1
2-maleylacetate	$C_6H_4O_5$	0.64	[M-2H]+	157.01305	3
Adenosine 5'-monophosphate	$C_{10}H_{14}N_5O_7P$	0.64	[M+H]+	348.06929	1
Nicotinic acid	$C_6H_5NO_2$	0.64	[M+H]+	124.03988	1
Methionine	$C_5H_{11}NO_2S$	0.64	[M+H]+	150.0567	1
Succinic acid	$C_4H_6O_4$	0.65	[M+H]+	119.03968	1
Aminoundecanoic acid	$C_{11}H_{23}NO_2$	0.66	[M+H]+	202.17957	3
Uridine	$C_9H_{12}N_2O_6$	0.66	[M+H]+	245.07566	1
Adenine	$C_5H_5N_5$	0.66	[M+H]+	136.06284	1
Nicotinamide	$C_6H_6N_2O$	0.67	[M+H]+	123.04904	1
L-Tyrosine	$C_9H_{11}NO_3$	0.68	[M-NH$_2$]+	165.05422	1
L-Tyrosine	$C_9H_{11}NO_3$	0.68	[M+H]+	182.08059	1
Adenosine	$C_{10}H_{13}N_5O_4$	0.69	[M+H]+	268.10259	1
Tyramine	$C_8H_{11}NO$	0.73	[M-NH$_2$]+	121.06483	1
Guanosine	$C_{10}H_{13}N_5O_5$	0.74	[M+H]+	284.09886	2
Tyramine	$C_8H_{11}NO$	0.74	[M+H]+	138.08954	1
Inosine	$C_{10}H_{12}N_4O_5$	0.76	[M+H]+	269.09141	1
Hypoxanthine	$C_5H_4N_4O$	0.76	[M+H]+	137.04988	1
Gamma-methyl L-glutamate	$C_6H_9NO_3$	0.92	[M-H$_2$O+H]+	144.06494	3
L-Phenylalanine	$C_9H_{11}NO_2$	1.21	[M+H]+	166.08536	1
Phenylalanine	$C_9H_{11}NO_2$	1.23	[M-CH$_2$O+H]+	120.08096	1
L-Phenylalanine	$C_9H_{11}NO_2$	1.3	[M+CH$_2$O$_2$Na]+	234.07395	1
Phenylalanylglutamate	$C_{14}H_{18}N_2O_5$	1.47	[M+H]+	295.13017	3
D-pantothenic acid	$C_9H_{17}NO_5$	1.59	[M+H]+	220.11212	1
HDMBOA, 2-Hydroxy-4,7-dimethoxy-2H-1,4-benzoxazin-3(4H)-one	$C_{10}H_{11}NO_5$	1.64	[M+H]+	226.0706	3
Protocatechuic acid	$C_7H_6O_4$	1.79	[M+H]+	155.03549	1
L-Tryptophan	$C_{22}H_{24}N_4O_4$	2.33	[2M+H]+	409.1869	1
3,4-Dimethoxyphenethylamine	$C_{10}H_{15}NO_2$	2.34	[M+H]+	182.11745	1
Tryptophan, in-source fragment	$C_{11}H_{12}N_2O_2$	2.35	[M-CHO$_2$]+	159.09081	1
Tryptophan, in-source fragment	$C_{11}H_{12}N_2O_2$	2.36	[M-C$_2$H$_4$NO]+	146.06003	1
L-Tryptophan	$C_{11}H_{12}N_2O_2$	2.37	[M+H]+	205.09657	1
5-S-Methyl-5-thioadenosine	$C_{11}H_{15}N_5O_3S$	2.48	[M+H]+	298.09673	3
Gentisic acid	$C_7H_6O_4$	2.52	[M-H$_2$O+H]+	137.0248	1
Anthranilate	$C_7H_7NO_2$	2.52	[M+H]+	138.05398	3
Leucyl-Proline	$C_{11}H_20N_2O_3$	2.57	[M+H]+	229.15289	1
Esculin	$C_{15}H_{16}O_9$	2.71	[M+H]+	341.08723	1
3-Amino-4-hydroxybenzoic acid, 5-amino saliciylic acid	$C_7H_7NO_3$	2.77	[M+H]+	154.04987	3
2-Hydroxybenzoic acid, Salicylic acid	$C_7H_6O_3$	2.78	[M-H$_2$O+H]+	121.02945	1
Tryptamine	$C_{10}H_{12}N_2$	2.79	[M+H]+	161.10689	1

2,4 Dihydroxybenzoic acid	$C_7H_6O_4$	2.9	[M-H_2O+H]+	137.02384	2
β-Carbocine-3-carboxylic acid	$C_{12}H_{12}N_2O_2$	3	[M+H]+	217.09646	2
Hydroxycoumarin 1	$C_9H_6O_3$	3.09	[M+H]+	163.04201	3
Methyl 4-methoxycinnamate	$C_{11}H_{12}O_3$	3.15	[M+H]+	193.0832	3
ubiquitous metabolite	$C_{11}H_{28}N_2O_{12}$	3.17	[M+H]+	381.17402	4
Scopoletin hexose-pentose	$C_{21}H_{26}O_{13}$	3.19	[M+H]+	487.14409	3
Scopolin	$C_{16}H_{18}O_9$	3.2	[M+H]+	355.10117	1
L-Tyrosyl-L-leucine	$C_{15}H_{22}N_2O_4$	3.27	[M+H]+	295.17048	2
Quercetin-3-O-glc-1-3-rham-1-6-glucoside	$C_{33}H_{40}O_{21}$	3.33	[M+H]+	773.20921	3
Fraxin	$C_{16}H_{18}O_{10}$	3.42	[M-$C_6H_{10}O_5$+H]+	209.0463	1
Derivative of dicaffeoyl quinic acid	$C_{24}H_{18}O_{12}$	3.57	[M+H]+	499.087	3
Unknown Hexoside	$C_{19}H_{30}O_8$	3.64	[M+H]+	387.20108	3
7,8-Dihydroxycoumarin	$C_9H_6O_4$	3.67	[M+H]+	179.03724	1
9,10-Dihydrohydroxy-jasmonic acid sulfate	$C_{12}H_{20}O_7S$	3.68	[M+H]+	309.10273	2
Riboflavin	$C_{17}H_{20}N_4O_6$	3.71	[M+H]+	377.14348	1
β-d-glucosyl indole-3-carboxylate	$C_{15}H_{17}NO_7$	3.85	[M+H]+	324.10978	2
Quercetin + Hex + Hex + Fuc	$C_{33}H_{40}O_{21}$	3.92	[M+H]+	773.20551	3
Quercetin + Hex+Hex	$C_{27}H_{30}O_{17}$	3.96	[M+H]+	627.1529	3
Quercetin Deoxyhex Deoxyhex Hex	$C_{33}H_{40}O_{20}$	4.01	[M+H]+	757.21447	3
Saponarin	$C_{27}H_{30}O_{15}$	4.01	[M+H]+	595.16569	2
Quercetin Deoxyhex Hex	$C_{27}H_{30}O_{16}$	4.02	[M+H]+	611.15714	3
Isoorientin	$C_{21}H_{20}O_{11}$	4.02	[M+H]+	449.10799	3
1-O-Feruloyl-beta-D-glucose	$C_{16}H_{20}O_9$	4.06	[M+H]+	357.11916	3
N-Phenylacetyl-Aspartic acid	$C_{12}H_{13}NO_5$	4.07	[M+H]+	252.0869	2
Hydroxyjasmonic acid	$C_{12}H_{18}O_4$	4.11	[M+H]+	227.12685	3
Sinapyl alcohol	$C_{11}H_{12}O_3$	4.17	[M-H_2O+H]+	193.08493	1
Phthalide	$C_8H_6O_2$	4.18	[M+H]+	135.04479	1
Leucyl-Phenylalanine	$C_{15}H_{22}N_2O_3$	4.19	[M+H]+	279.16744	1
Carveol	$C_{10}H_{16}O$	4.2	[M+H]+	135.11612	3
Quercetin+hex+deoxyhex	$C_{27}H_{30}O_{16}$	4.23	[M+H]+	611.15799	3
fragment of Quercetin+hex+deoxyhex	$C_{21}H_{20}O_{12}$	4.24	[M+H]+	465.10365	3
fragment of Quercetin+hex+deoxyhex	$C_{15}H_{10}O_7$	4.24	[M+H]+	303.0499	3
Unknown, similar to Tyrosine	$C_9H_{11}NO_3$	4.26	[M+H]+	182.08109	4
Unknown, similar to Feruloyl glycerol	$C_{13}H_{16}O_6$	4.29	[M+H]+	269.10204	4
Methylumbelliferone	$C_{10}H_8O_3$	4.39	[M+H]+	177.05444	3
Feruloyl dehydrotyramine	$C_{18}H_{17}NO_4$	4.4	[M+H]+	312.12362	3
Scopoletin	$C_{10}H_8O_4$	4.41	[M+H]+	193.05111	1
Methylumbelliferone	$C_{10}H_8O_3$	4.44	[M+H]+	177.05434	3
Rutin, Quercetin 3-o-rutinoside	$C_{27}H_{30}O_{16}$	4.45	[M+H]+	611.15984	1
Rutin, in-source fragment 302	$C_{15}H_{10}O_7$	4.45	[M+H]+	303.0498	1
Rutin, in-source fragment 464	$C_{21}H_{20}O_{12}$	4.45	[M+H]+	465.10349	1
Gibberellic acid	$C_{19}H_{18}O_4$	4.49	[M-H_4O_2+H]+	311.12722	1
Leuko-DOPA-chrome	$C_9H_9NO_4$	4.56	[M+H]+	196.06113	3
Quercetin-3-o-glucoside, in-source fragment -Glc	$C_{15}H_{10}O_7$	4.59	[M+H]+	303.04987	1

Quercetin-3-glucoside, Isoquercitrin	$C_{21}H_{20}O_{12}$	4.59	[M+H]+	465.10251	1
Benzoic acid	$C_7H_6O_2$	4.64	[M+H]+	123.04509	1
N-(3-Hydroxypropyl)-phthalimide; 1H-Isoindole-1,3(2H)-dione	$C_{11}H_{11}NO_3$	4.76	[M+H]+	206.0799	1
Guaiacylglycerol β-coniferyl ether (G(8-O-4)G)	$C_{20}H_{24}O_7$	4.81	[M+H]+	377.16009	3
Kaempferol 3-O-beta-Glc-7-O-alpha-Rha	$C_{27}H_{30}O_{15}$	4.81	[M+H]+	595.1655	2
4-Hydroxy-3,5-dimethylbenzoic acid	$C_9H_{10}O_3$	4.85	[M+H]+	167.07077	2
Unknown, similar to Tyrosine	$C_9H_{11}NO_3$	4.86	[M+H]+	182.08115	4
5-Methoxyindole-3-acetic acid	$C_{11}H_{11}NO_3$	4.98	[M+H]+	206.07929	1
Unknown polysaccharide	$C_{12}H_{22}O_{11}$	5.31	[M+H]+	365.10049	4
Kaempferin	$C_{21}H_{20}O_{10}$	5.41	[M+H]+	433.1236	3
3,4-dimethoxycinnamic acid	$C_{11}H_{12}O_4$	5.6	[M+H]+	209.08191	1
Simulanol (S(8-5)G)	$C_{21}H_{24}O_7$	5.67	[M+H]+	389.16803	2
N-feruloyltyramine	$C_{18}H_{19}NO_4$	5.84	[M+H]+	314.13753	2
Glyphosate constituent 1	$C_{16}H_{33}NO_3$	5.89	[M+H]+	288.2528	4
Abscisic acid	$C_{15}H_{18}O_3$	5.9	[M+H]+	247.13147	1
Glycosmisic acid	$C_{20}H_{20}O_7$	5.91	[M+H]+	373.13667	2
p-Coumaric acid-O-dihydroxy-cinnamoyl methyl ester	$C_{19}H_{16}O_6$	5.92	[M+H]+	341.10174	3
Glyphosate constituent 2	$C_{16}H_{33}NO_3$	6.06	[M+H]+	288.25272	4
(E)-Cinnamic acid	$C_9H_8O_2$	6.09	[M+H]+	131.04966	1
Herniarin	$C_{10}H_8O_3$	6.17	[M+H]+	177.05415	1
Glyphosate constituent 3	$C_{16}H_{33}NO_3$	6.3	[M+H]+	288.25232	4
Amaranthus triterpenoid, 1258 (fragment 502)	$C_{30}H_{46}O_6$	6.64	[M+H]+	503.33567	3
Genistein	$C_{15}H_{10}O_5$	6.77	[M+H]+	271.06077	1
Amaranthus triterpenoid, 792	$C_{40}H_{56}O_{16}$	6.79	[M+H]+	793.36031	3
Amaranthus triterpenoid, 840 (fragment 502)	$C_{30}H_{46}O_6$	6.79	[M+H]+	503.33582	3
Jasmonic acid	$C_{12}H_{18}O_3$	6.82	[M+H]+	211.13316	1
Amaranthus triterpenoid, 840	$C_{41}H_{60}O_{18}$	6.82	[M+H]+	841.38418	3
Amaranthus triterpenoid, 1216 (fragment 810)	$C_{40}H_{58}O_{17}$	6.89	[M+H]+	811.38031	3
Amaranthus triterpenoid, 1216 (fragment 764)	$C_{39}H_{56}O_{15}$	6.89	[M+H]+	765.37209	3
Amaranthus triterpenoid, 1216 (fragment 810)	$C_{40}H_{58}O_{17}$	6.98	[M+H]+	811.37071	3
Amaranthus triterpenoid, 1216 (fragment 502)	$C_{30}H_{46}O_6$	6.98	[M+H]+	503.3365	3
Farnesal	$C_{15}H_{24}O$	7.16	[M+H]+	203.17828	2
Amaranthus triterpenoid, 794 (fragment 486)	$C_{30}H_{46}O_5$	7.26	[M+H]+	487.33998	3
Amaranthus triterpenoid, 794 (fragment 308)	$C_{10}H_{12}O_{11}$	7.27	[M+H]+	309.04793	3
Amaranthus triterpenoid, 1405 (fragment 808)	$C_{41}H_{60}O_{16}$	7.59	[M+H]+	809.39309	3
Amaranthus triterpenoid, 824 (fragment 780)	$C_{40}H_{60}O_{15}$	7.84	[M+H]+	781.39422	3
Biochanin A, 5,7-Dihydroxy-4'-methoxyisoflavone (internal standard)	$C_{16}H_{12}O_5$	8.79	[M+Na]+	307.05808	1
Biochanin A, 5,7-Dihydroxy-4'-methoxyisoflavone (internal standard)	$C_{16}H_{12}O_5$	8.79	[M+H]+	285.07614	1
Phytosphingosine	$C_{18}H_{37}NO_3$	9.32	[M+H]+	316.28323	3
Methyl octanoate	$C_9H_{18}O_2$	9.35	[M+H]+	159.13763	3
Dioscin	$C_{39}H_{63}O_{12}$	9.7	[M+H]+	724.44094	3
Lauric acid diethanolamide	$C_{16}H_{33}NO_3$	9.72	[M+H]+	288.25262	3
Glc-Glc-octadecatrienoyl-sn-glycerol (isomer 1)	$C_{33}H_{56}O_{14}$	9.8	[M+H]+	659.36079	3

Monolinolenin (isomer 1)	$C_{21}H_{36}O_4$	9.8	[M+H]+	353.26758	3
Glc-octadecatrienoyl-sn-glycerol (isomer 1)	$C_{27}H_{46}O_9$	10.03	[M+H]+	515.31914	3
Monolinolenin (isomer 2)	$C_{21}H_{36}O_4$	10.03	[M+H]+	353.26763	3
Glc-Glc-octadecatrienoyl-sn-glycerol (isomer 2)	$C_{33}H_{56}O_{14}$	10.04	[M+H]+	699.35227	3
Oxo-phytodienoic acid	$C_{18}H_{28}O_3$	10.24	[M+H]+	315.1929	2
Capric acid	$C_{10}H_{20}O_2$	10.32	[M+H]+	173.15204	1
Lysophosphatidylcholine (isomer 1)	$C_{26}H_{50}NO_7P$	10.62	[M+H]+	520.33946	3
Glc-octadecatrienoyl-sn-glycerol (isomer 2)	$C_{27}H_{46}O_9$	10.75	[M+H]+	497.31063	3
Linolenoylglycerol	$C_{21}H_{36}O_4$	10.75	[M+H]+	353.26762	3
Lysophosphatidylcholine (isomer 2)	$C_{26}H_{50}NO_7P$	10.84	[M+H]+	520.33978	3
Lysophosphatidylcholine (isomer 3)	$C_{24}H_{50}NO_7P$	11.03	[M+H]+	496.34017	3
Hydroxyoctadecadienoic acid (isomer 1)	$C_{18}H_{32}O_3$	11.33	[M+H]+	319.22361	3
Lysophosphatidylcholine (isomer 4)	$C_{26}H_{52}NO_7P$	11.44	[M+H]+	522.35566	3
1-Oleoyl-sn-glycero-3-phosphocholine	$C_{26}H_{52}NO_7P$	11.67	[M+H]+	522.35557	3
Hydroxyoctadecadienoic acid (isomer 2)	$C_{18}H_{30}O_3$	11.68	[M+H]+	277.21434	3
Lauric acid	$C_{12}H_{24}O_2$	12.09	[M+H]+	201.18435	1
Phthalic anhydride	$C_8H_4O_3$	12.2	[M+H]+	149.02348	3
1-Stearoyl-2-hydroxy-sn-glycero-3-phosphocholine	$C_{26}H_{54}NO_7P$	12.7	[M+H]+	524.37025	3
1-Stearoyl-sn-glycero-3-phosphate	$C_{21}H_{41}O_7P$	13.64	[M+H]+	437.2642	3
Linoloylglycerol	$C_{21}H_{38}O_4$	13.7	[M+H]+	377.26543	3
Oxylipin	$C_{36}H_{60}O_4$	13.72	[M+H]+	557.45365	3
linolenic acid (isomer1)	$C_{18}H_{30}O_2$	13.72	[M+H]+	279.23108	3
monoglyceride	$C_{20}H_{34}O_3$	13.89	[M+H]+	345.23948	3
linolenic acid (isomer 2)	$C_{18}H_{30}O_2$	13.89	[M+H]+	279.22526	3
13-apo-β-carotenone	$C_{18}H_{26}O$	13.97	[M+H]+	259.20489	3
Oleamide	$C_{18}H_{35}NO$	14.31	[M+H]+	282.27812	3
1-Palmitoylglycerol	$C_{19}H_{38}O_4$	14.36	[M+H]+	353.26486	3
17-Octadecynoic acid	$C_{18}H_{32}O_2$	14.58	[M+H]+	263.23577	3
1-Stearoyl-rac-glycerol	$C_{21}H_{42}O_4$	15.83	[M+H]+	359.31449	3
Diacylglycerine	$C_{39}H_{64}O_5$	17.82	[M+H]+	613.481	3

Reference: Matern A, Böttcher C, Eschen-Lippold L, Westermann B, Smolka U, Döll S, Trempel F, Aryal B, Scheel D, Geisler M, Rosahl S (2019). A substrate of the ABC transporter PEN3 stimulates bacterial flagellin (flg22)-induced callose deposition in Arabidopsis thaliana. J Biol Chem. 294(17):6857-6870. doi: 10.1074/jbc.RA119.007676.

Figure S1. Amino acids detected by GC-MS in glyphosate-resistant and sensitive *Amaranthus palmeri* plants . Y-axis: normalized peak intensity. Plants were untreated (control) or treated with glyphosate in two different doses: 0.25 RD (field recommended dose) or RD in GS and RD or 3 RD in GR. Mean ± SE (n=4-6). Different letters within each population indicate significant differences between treatments (p-value≤0.05, Tukey).

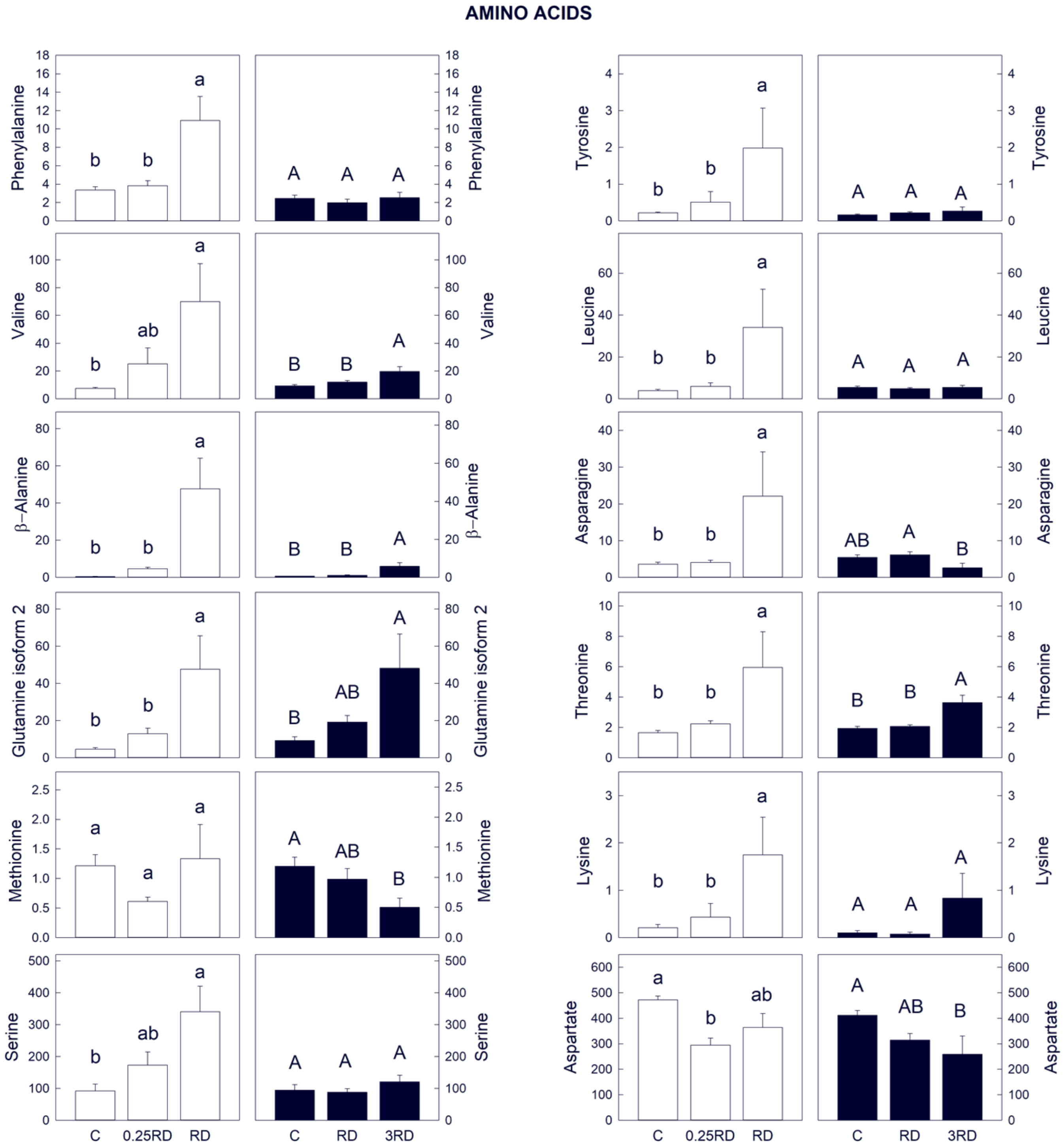
AMINO ACIDS
Phenylalanine
Tyrosine
Valine
Leucine
β−Alanine
Asparagine
Glutamine isoform 2
Threonine
Methionine
Lysine
Serine
Aspartate
C
0.25RD
RD
C
RD
3RD

Figure S2. A. Treatment application with an aerograph connected to a compressor. **B.** State of the plant at the moment of the treatment.

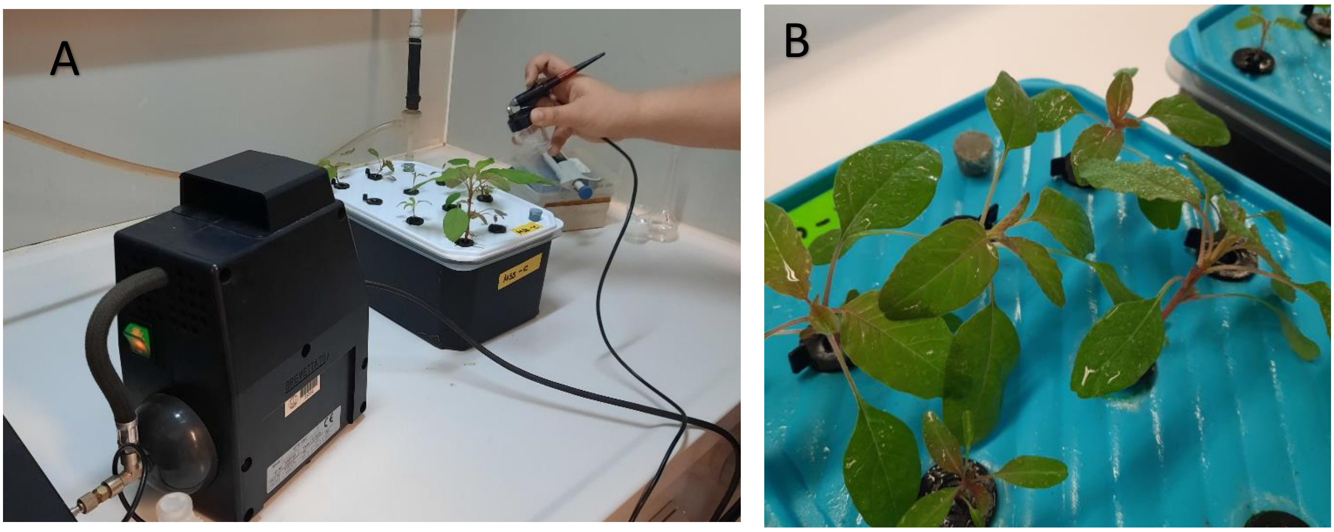

Supplement: Supplementary file 1 [file plants-12-01345-s001.zip › plants-2105890-supplementary.pdf]
